# Interleukin-15 Constrains Mucosal T Helper 17 Cell Generation: Influence of Mononuclear Phagocytes

**DOI:** 10.1371/journal.pone.0143001

**Published:** 2015-11-23

**Authors:** Huifeng Yu, Yongjun Sui, Yichuan Wang, Noriko Sato, Blake Frey, Zheng Xia, Thomas A. Waldmann, Jay Berzofsky

**Affiliations:** 1 Vaccine Branch, Center for Cancer Research, National Institute of Health, Bethesda, Maryland, United States of America; 2 Molecular Imaging Program, Center for Cancer Research, National Cancer Institute, National Institute of Health, Bethesda, Maryland, United States of America; 3 Metabolism Branch, Center for Cancer Research, National Cancer Institute, National Institute of Health, Bethesda, Maryland, United States of America; Jackson Laboratory, UNITED STATES

## Abstract

Interleukin (IL)-15 has multiple roles in innate and adaptive immunity, especially regarding CD8^+^ T cells and natural killer cells. However, the role of IL-15 in regulating differentiation of T helper cell subsets and mononuclear phagocytes (MPs) in different tissues *in vivo* is unknown. Here we report that IL-15 indirectly regulates Th17 but not other Th subsets in the intestinal lamina propria (LP), apparently through effects on MPs. Th17 cells in the LP were more prevalent in IL-15 KO mice than their wild-type counterparts, and less prevalent in IL-15 transgenic mice than their wild-type littermates, even co-caged. MPs from the LP of these mice were sufficient to mimic the *in vivo* finding *in vitro* by skewing of cocultured wild type OVA-specific CD4^+^ T cells. However, production of IL-15 or lack thereof by these MPs was not sufficient to explain the skewing, as addition or blockade of IL-15 in the cultures had no effect. Rather, a skewing of the relative proportion of CD11b^+^, CD103^+^ and double positive LP MP subsets in transgenic and KO could explain the differences in Th17 cells. Thus, IL-15 may influence MP subsets in the gut in a novel way that alters the frequency of LP Th17 cells.

## Introduction

The cytokine interleukin 15 (IL-15), a protein of 114 amino acids, was first discovered in 1994 and had IL-2 like stimulatory actions on T cells [1, 2]. It is a pleiotropic cytokine of the common cytokine receptor γ chain family, which includes IL-2, IL-4, IL-7, IL-9 and IL-21 [3, 4]. IL-15 is produced by a broad array of cell types, which includes dendritic cells (DCs), monocytes, epithelial cells, macrophages, and fibroblasts [5].

Remarkable progress has been made in understating of IL-15 biology, including its role in the normal host immune responses and its potential for participation in the pathogenesis of disease since its discovery [5]. IL- 15 has multiple roles in the innate and adaptive immune system, including the development, activation, homing and survival of immune effector cells, especially CD8^+^ T cells, natural killer cells and natural killer T cells. In light of the crucial role of IL-15 in the generation and maintenance of these immune cells, using IL-15 as an adjuvant provides a new perspective for the development of preventive vaccines against tumors and infectious agents [6–12]. Conversely, IL-15 is a pro-inflammatory cytokine and plays a primary role in the development of autoimmune diseases and inflammatory diseases such as rheumatoid arthritis, sarcoidosis, inflammatory bowel disease [5].

The receptor of IL-15 is a heterotrimeric receptor composed of IL-15R α, IL-2/IL-15R β and γ chain. IL-15R α alone is sufficient for high affinity binding of IL-15 and can present IL-15 *in trans* to cells that express IL-2/IL-15Rβ and γ chain but not IL-15Rα [13, 14]. IL-2/IL-15Rβ interacts with JAK1, and the γ chain with JAK3 and together lead to phosphorylation of STAT-5 and STAT3, which affect cellular survival and proliferation, and also through β chain interaction with Shc induce the MAP kinase and PI3 kinase/AKT pathways that lead to mitogenic and antiapoptotic signals [7, 15].

Naïve CD4^+^ T cells can differentiate, during a primary antigen response, into several distinct polarized subsets such as Th1, Th2, regulatory T cells (Tregs), as well as the more recently discovered lineage Th17 cells [16, 17]. Th1 cells mainly produce IFNγ, which is important for macrophage activation and clearance of intracellular pathogens, whereas Th2 cells produce IL-4, IL-5 and are critical for clearance of extracellular parasites [18]. Natural regulatory T cells (nTregs) develop in the thymus and are responsible for immunologic self-tolerance and negative control of immune responses [19]. Th17 cells producing IL17 play important roles during immune responses against extracellular bacteria and fungi, and are involved in autoimmune diseases [20]. Earlier studies support the classification of IL-15 as a proinflammatory type-1 cytokine [21–23], whereas a few have observed IL-15 as a costimulator of type-2 cytokines [24]. The addition of exogenous IL-15 favored human Th1 T cell differentiation *in vitro* [22]. These data suggested that the role of IL-15 in the development of CD4^+^ T cell immunity is complex. However, the role of IL-15 in CD4^+^ T helper cell differentiation at the level of the whole organism by using IL-15 deficient mice and IL-15 transgenic (Tg) mice has not been studied. Our present study addresses this issue.

Mononuclear phagocytes (MPs) that function as antigen presenting cells (APC), especially dendritic cells (DCs) and macrophages, are essential for the different lineages of CD4^+^ T cell polarization. MPs expressing CD11c and MHC II were originally believed to be just DCs, but more recent evidence suggests that macrophages in the gastrointestinal tract can express CD11c, causing some confusion in the classification of intestinal lamina propria (LP) MPs [25–28]. Classification has usually been based on the expression of CD11b, CD8α, and CD103, defining distinct subsets of CD103^+^CD11^-^, CD103^+^CD11b^+^ and CD103^-^CD11b^+^ MPs that exhibit different functional properties [29–32]. Both subsets that express CD103 are generally believed to be true DCs, whereas some of the CD103-negative subsets, including many CD11b single-positive cells and especially those expressing CX3CR1 (which extend dendrites through the epithelium but now appear to be sessile macrophages), include macrophages derived from Ly6C^+^ monocytes rather than from DC precursors [25–28]. However, recently a population of CD103^-^ CD11b^+^ cells was defined that are true DCs, lacking the macrophage markers CD64 and F4/80 [25, 33]. While sessile macrophages may not be able to traffic to draining lymph nodes (LNs) to prime T cells there, these CD11b^+^ single positive true DCs can traffic to the draining LNs and thus prime T cells and skew their phenotype. CD103^+^ MPs could induce gut-homing molecules on effector T cells and help the generation of Treg cells [34–36]. CD8α^+^ DCs and CD103^+^CD11b^-^ DCs are especially effective at cross-presentation to CD8^+^ T cells [25, 27]. The CD103^+^CD11b^+^ (double positive) MP subset and CD103^-^CD11b^+^ single positive MPs promote Th17 cell differentiation either constitutively or in responses to TLR ligands [25–28, 37–42]. A newly defined subset of CD103-negative CD11b^+^ true DCs that express CCR2 has been found to be especially effective at inducing Th17 differentiation (even more so than double positive MPs) [33]. Thus, the IL-17-inducing MP subsets are generally those expressing CD11b, with or without CD103. Both of these IL-17-inducing CD11b^+^ subsets were found to be IRF4-dependent [25, 33]. Moreover, the tissue environment in which MPs reside has a major impact on their phenotypic and functional properties [43]. Besides effects of the microbiome [44], vitamin A (retinol) from diet or from bile can influence MP imprinting within the intestinal mucosa [45].

IL-15 knockout (KO) mice have been shown to have reduced numbers of memory CD8^+^ T cells, NKT cells, NK cells, and subsets of intestinal intraepithelial lymphocytes [46]. IL-15 receptor deficient mice also demonstrate a broadly similar phenotype [47]. On the other hand, IL-15 Tg mice have early expansion of CD8^+^ T cells and NK cells [5, 48] and unconventional CD8αα NK1.1^+^ T cells [49]. However, the distribution of MP subsets in these mice and their role in the differentiation of CD4^+^ T cell polarized subsets remains unexplored. Thus, here we address two previously unaddressed questions, the role of IL-15 in differentiation of CD4^+^ helper T cell subsets in the gut mucosa and its role in the differentiation of MP subsets in the gut mucosa, and the connection between these.

## Material and Methods

### Mice

Six to eight week old C57BL/6 mice or C57BL/6 background IL-15 KO mice and OT-II mice were purchased from Taconic Farms (New York, USA). Human IL-15 gene Tg mice were previously reported [48]. Mice were maintained in specific pathogen free conditions in the animal facility of National Cancer Institute. All animal protocols were reviewed and approved by the Animal Care and Use Committee of the National Institutes of Health. Mice to be compared were co-housed in the same cage to avoid differences in their gut microbiome.

### Antibodies and reagents

FITC or PE-Cy5 conjugated anti-mouse CD11b (clone M1/70), Percp or PE-Cy7 conjugated anti-mouse CD11c (clone N418), APC or Percp conjugated anti-mouse CD4 (clone GK1.5), PE or FITC conjugated anti-mouse CD3 (clone 17A2), APC conjugated anti-mouse IFNγ (clone XMG1.2), APC or PE conjugated anti-mouse IL-17A (clone TC11-18H10.1), APC or FITC conjugated anti-mouse Foxp3 (clone MF14), APC conjugated anti-mouse CD103 (clone 2E7), and Pacific Blue conjugated anti-mouse MHC class II antibodies were purchased from Biolegend (San Diego, CA, USA). PE conjugated anti-mouse CD8α (clone 53–6.7), FITC or APC conjugated anti-mouse MHC class II (clone M5/114.15.2), APC conjugated anti-mouse CD11b (clone M1/70) and purified anti-mouse CD3 antibodies were from eBioscience (San Diego, CA, USA). PE conjugated anti-mouse CD103 (M290) was purchased from BD Bioscience. Recombinant human IL-15, recombinant mouse TGF-β, GM-CSF and IL-6, rabbit anti-human and anti-mouse IL-15 were purchased from PeproTech (Rocky Hill, NJ, USA). Stained cells were analyzed on FACSCalibur or LSRII flow cytometers (BD Biosciences).

### Isolation of cells

For splenocytes, spleens were cut into small fragments and then were digested for 30 min at 37°C with 2mg/ml collagenase D (Sigma) in complete culture medium. For cell from draining LNs, LNs were directly minced and cell suspensions were passed through a strainer. For cells from the small intestine, small intestines were removed and were carefully cleaned of their mesentery, then Peyer’s patches were excised and the intestines were opened longitudinally and washed of fecal contents. Intestines were cut into 1.0 cm pieces, which were incubated and shaken in Hanks’ balanced salt solution containing 5mM EDTA and 0.1mM dithiothreitol for 20 min at 37°C to separate intraepithelial lymphocytes (IEL). Cell suspensions (IEL) were passed through a strainer and purified on a cushion of Lympholyte-M separation medium. The remaining intestinal tissue was washed, then minced, and then transferred to a 50 ml conical tube and incubated for one hour in complete culture medium containing 2 mg/ml collagenase D and 200 μg/ml DNAse I (Sigma). Cell suspensions (lamina propria) were collected and passed through a strainer and pelleted by centrifugation at 300 x g. The cells were resuspended in 10 ml of the 40% Percoll and separation was performed by centrifugation for 10 min at 800 x g at room temperature. The cells were resuspended and washed twice for different experiments.

### Culture of mononuclear phagocytes (MPs) with CD4^+^ T cells

MPs from spleen or small intestine lamina propria (LP) were enriched by positive immunomagnetic selection using anti-mouse CD11c beads (Miltenyi Biotec). OTII CD4+ T cells were isolated from splenocytes using a CD4^+^CD62L^+^ T cell isolation kit II from Miltenyi Biotec. MPs were shown to be >90% CD11c^+^ and 99% MHC II^+^ (S2 Fig).

For *in vitro* stimulation, purified LP MPs (1 x 10^5^) were cultured together with OTII CD4^+^ T cells at a ratio of 1:2 in 250 μl RPMI complete medium in 96 well round-bottomed plates. For antigen specific Th17 differentiation, 5 μg/ml OVA_323–339_ (ISQAVHAAHAEINEAGR) was added to the MP and OTII CD4^+^ T cell coculture experiments in the presence or absence (as a control) of IL-6 (10ng/ml), TGF-β (5ng/ml), and anti-IFNγ (50μg/ml). In some experiments, human IL-15 (which acts on mouse cells) at 20 ng/ml or anti-mouse IL-15 at 5 μg/ml or anti-human IL-15 at 5 μg/ml (which both block mouse IL-15) were added in the co-culture of OT II CD4^+^ T cells and wild type (WT) MPs to induce Th17 cells. After 4 days, the cocultures were restimulated for 4 hours with 20 ng/ml phorbol 12-myristate 13-acetate (PMA) and 1 μg/ml ionomycin in the presence of brefeldin A for intracellular cytokine staining.

### Intracellular staining and cell surface staining

After cell surface staining with anti-CD4 and anti-CD3, cells were permeabilized with Cytofix/Cytoperm (PharMingen, San Diego, CA) in accordance with the manufacturer’s recommendations. Intracellular staining was performed with anti-IFNγ, anti-IL17A, and anti-Foxp3. Cells were analyzed on a fluorescence-activated cell sorter (FACS) Calibur (BD Biosciences) or LSR II and analyzed by using CellQuest software (BD Biosciences) or FlowJo software (TreeStar, San Carlos, CA).

### Statistics

Values are expressed as mean ± standard deviation (SD). A value of <0.05 indicates significance. Statistical analysis was performed using nonparametric Mann-Whitney test. A paired Student t-test was used for comparing different CD11b, CD103 subset MPs.

## Results

### Increased frequencies of Th17 cells in IL-15 deficient mice are consistent with reduced frequencies of Th17 in IL-15 transgenic mice

**Fig 1:**
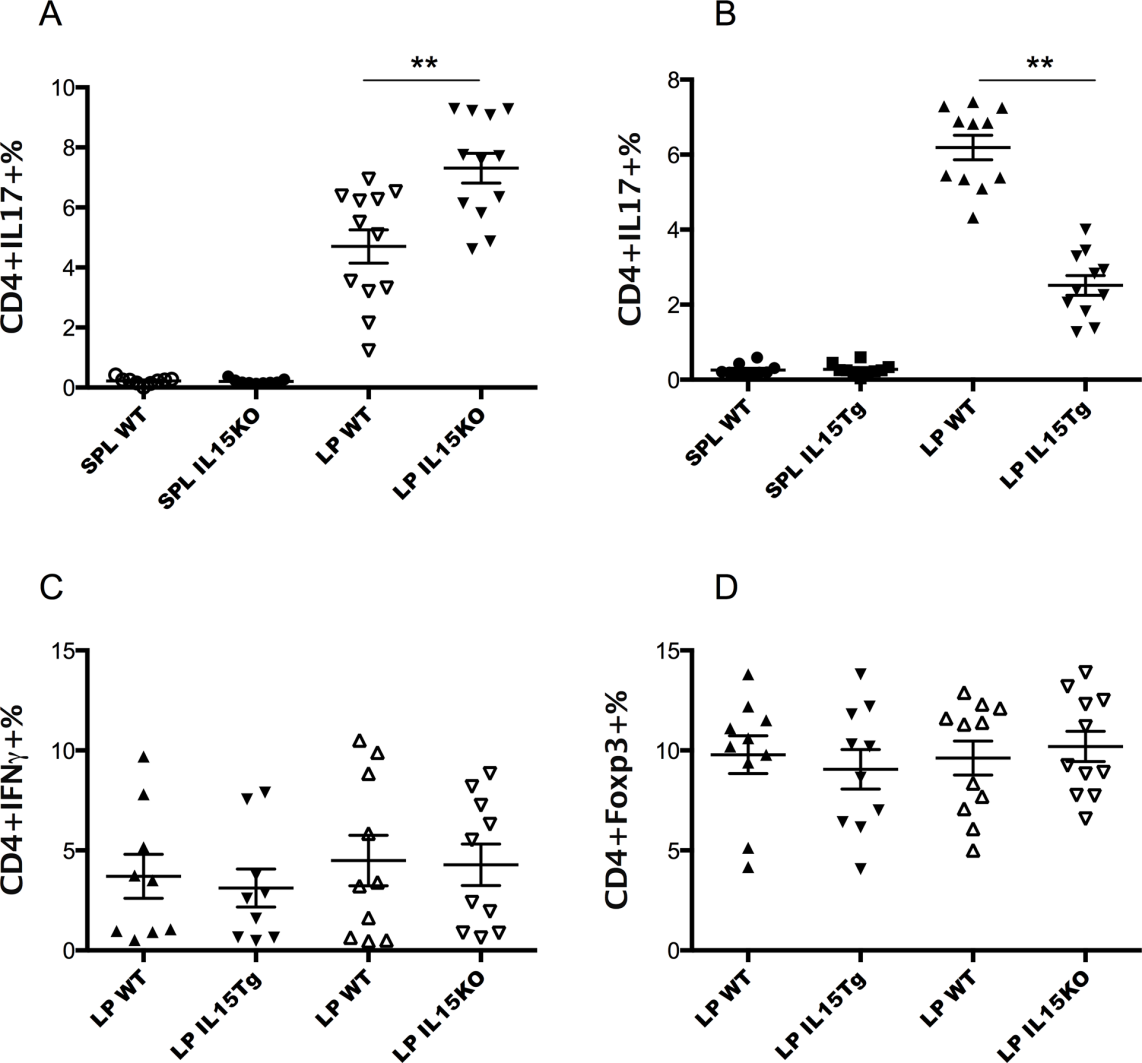
Differences in Th17 cell frequencies in the LP of IL-15 Tg or KO mice, but not in frequencies of Th1 or Treg cells. Cells from spleen and small intestine LP were stimulated for four hours with PMA and ionomycin and stained with surface markers CD3 and CD4, followed by intracellular staining of IL-17, IFNγ or Foxp3. **A.** Higher frequencies of Th17 cells were found in the LP of IL-15 KO mice than matched co-caged WT mice (p < 0.001 by Mann-Whitney). **B.** IL-15 Tg mice showed significantly lower frequencies of Th17 cells in the LP compared with their co-caged WT littermates (the littermates were considered as matched WT controls) (p < 0.001 by Mann-Whitney). **C.** The frequencies of IFNγ -producing CD4^+^ T cells were not significantly different in the LP of IL-15 KO *vs* WT and IL-15 Tg *vs* littermate WT control. **D.** The frequencies of CD3^+^CD4^+^Foxp3^+^ cells were not significantly different in small intestinal LP of IL-15 KO *vs* WT and IL-15 Tg *vs* littermate WT control. This figure pools data from several independent experiments with similar results. **D**ouble asterisk (**denotes P<0.001).

Th17 cells are a subset of effector CD4^+^ Th cells defined by their production of IL-17 [50]. Specific commensal microbiota play a critical role in Th17 cell differentiation in the LP of the small intestine [51]. In our present experiments, all the mice were co-caged to ensure similar gut microbiota. Here, we first examined Th17 frequency among CD3^+^CD4^+^ cells in the small intestine LP and spleen of IL-15 Tg mice and IL-15 KO mice as compared to their matched WT counterpart mice. In agreement with the literature, a large number of Th17 cells were specifically enriched in the small intestinal LP of normal naïve un-manipulated mice [52]. In contrast, Th17 cells were hardly detectable in the spleens of these mice **(Fig 1A and 1B)**. Interestingly, IL-15 KO mice showed much higher frequencies of Th17 cells in the LP than WT mice, but not in the spleen (**Fig 1A**). In contrast, IL-15 Tg mice showed much lower frequencies of Th17 cells in the LP as compared with their littermates (the WT littermate was considered as WT control mice for IL-15 Tg mice, **Fig 1B**). These data suggest that loss of IL-15 in mice leads to an increase of Th17 cells, and excess IL-15 does the converse.

### Th1 and Treg cells showed no significant differences in LP of IL-15- deficient mice and IL-15 transgenic mice compared to their matched WT mice

Earlier studies support the classification of IL-15 as a proinflammatory type-1 cytokine [22, 53, 54]. Moreover, addition of exogenous IL-15 favored human Th1 T cell differentiation *in vitro* [22]. Here we investigated the frequencies of Th1 cells in these IL-15 KO and Tg mice. It was surprising to see that the frequencies of Th1 cells were not significantly different in the small intestinal LP among these mice (**Fig 1C),** in contrast to the finding for Th17 cells above.

The same was true, surprisingly, for Tregs as well, despite the fact that we found fewer Th17 cells in the small intestine LP of IL-15 Tg mice, and published evidence indicated reciprocal Th17 and Treg cell differentiation in the gut [55]. In addition, IL-15 has been shown to be a potent inducer of CD4^+^CD25^high^ cells expressing Foxp3 in humans and governs the CD4^+^Foxp3^+^ Treg cell development [56, 57]. Moreover, the common γ chain cytokine IL-2, which is a growth factor for Tregs, inhibits the generation of Th17 cells and promotes the generation of Tregs [58]. *Il2* ^*−/-*^ mice exhibit reduced numbers of Tregs, and have an increased frequency of Th17 cells in the peripheral repertoire. Therefore, it was surprising that no significant difference was observed in the frequencies of CD4^+^Foxp3^+^ Treg cells in the LP between IL-15 KO mice and WT mice or between IL-15 Tg mice and their WT littermates (**Fig 1D**). This may reflect differences between *in vitro* models and *in vivo* situations. *In vitro*, there is a true reciprocity between the Th17 and Treg developmental programs on the single-cell level [50]. However, this reciprocal developmental decision *in vivo* at the single-cell level is more complicated and could be interfered with by various factors [50]. Our current data do not support a critical role of IL-15 in Treg cell differentiation in the gut *in vivo*. A representative FACS plot for each cell type is shown S1 Fig.

### Phenotypic characterization of MPs in IL-15 deficient mice and IL-15 transgenic mice

**Fig 2:**
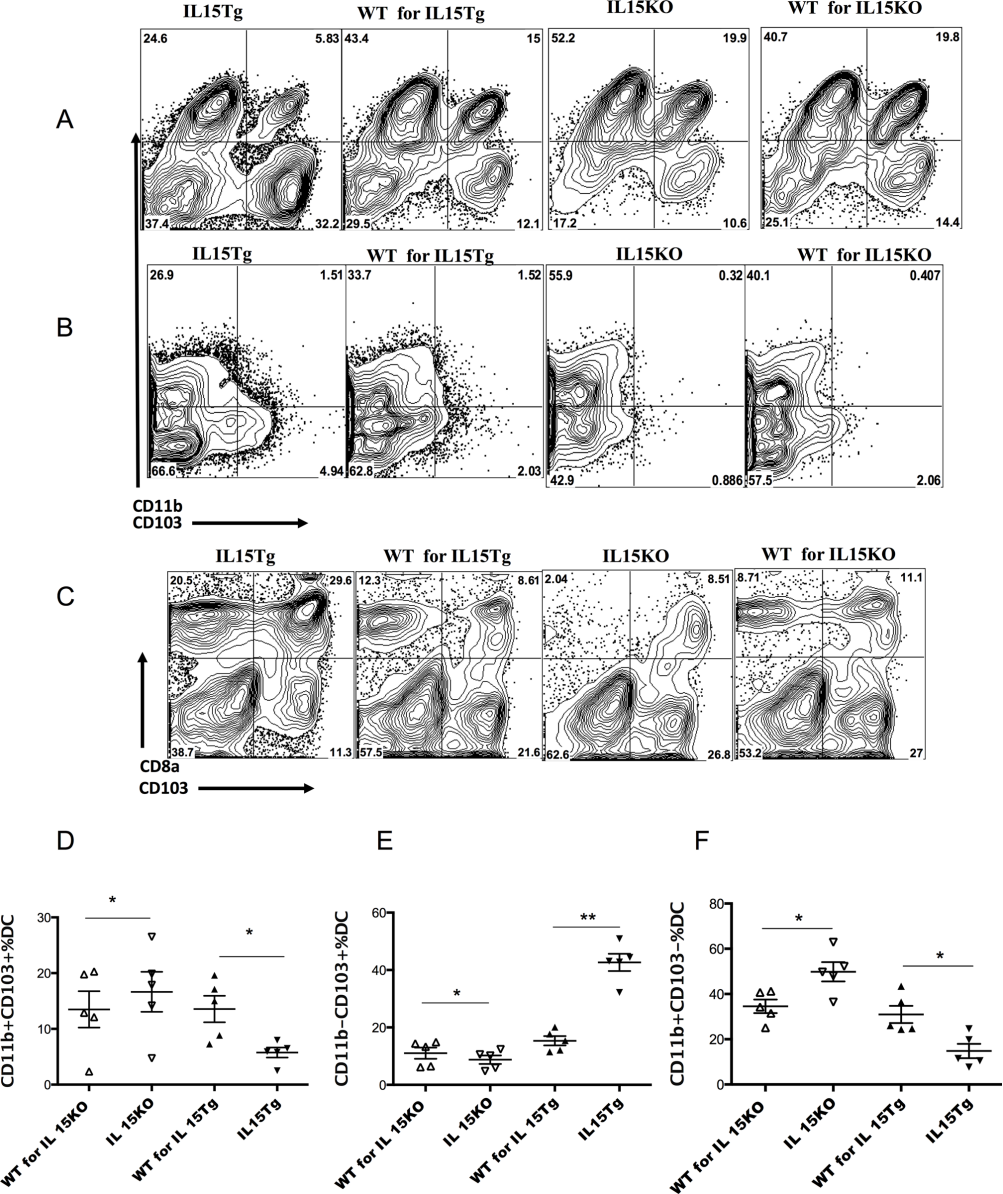
Characterization of MP subsets from intestinal LP and spleen. MPs were purified from small intestinal LP and spleen and then stained for surface markers CD103, CD11b, CD8, and MHCII. **A.** Purified MPs from intestinal LP were analyzed for CD11b and CD103 expression. One representative mouse was shown. **B.** Purified MPs from the spleen were analyzed for CD11b and CD103 expression. Far fewer CD103^+^ MPs were found in the spleen, as expected, but CD103 expression was higher in IL-15 Tg compared to WT mice. Conversely, CD11b expression was slightly lower in IL-15 Tg compared to WT, but slightly higher in the IL-15 KO *vs* WT. **C**. MPs purified from LP were analyzed for CD8α and CD103 expression. FACS plots are representative of five independent experiments of two mice pooled in each. The pooled data are shown in D, E and F. D. Percent of double positive CD11b and CD103 MP in the LP. E. Percent of single positive CD103 MPs. F. Percent of single positive CD11b MPs. The ratios of CD11b^+^CD103^+^/ CD103^+^ MPs (G) and CD11b^+^CD103^-^/ CD103^+^ MPs (H) based on purified LP cell preparations are shown in G and H. Paired Student t-tests were used for comparing different CD11b, CD103 subset MPs.

The influence of IL-15 could be direct or indirect, for example, through an effect on MP subsets. Published reports show that different MP subsets promote (e.g. CD11c^+^CD11b^+^ MPs) or inhibit (e.g. CD103^+^ CD11b^-^ MPs) differentiation of Th17 cells [38, 39, 59]. MPs expressing both CD11b and CD103 can also induce Th17 cells [25, 27, 40–42]. Thus, a common feature of the IL-17-inducing MPs is expression of CD11b, although not all CD11b^+^ MPs induce IL-17. Furthermore, in vitro, IL-15 has been shown to skew the development of MPs from monocytes to produce fewer of the CD11b^+^ MP subset [60]. Therefore, we asked whether the presence or absence of IL-15 affected MP subsets in the LP that could affect Th17 differentiation. We characterized MPs based on co-expression of CD11c and MHC II initially by gating (S3A–S3E Fig) and then, based on those results, in more detail using MPs purified with a Miltenyi MP isolation kit (Fig 2) (see S2 Fig). It is likely that CD11c^+^CD11b^+^CD103^-^ MPs contain some CD11c^+^ macrophages as well as DCs, although the macrophages tend to be less CD11c bright and are more prevalent in the colon [40]. Levels of CD11c and MHC II in our MP populations were heterogeneous but the majority were bright for both markers (S2 Fig). Nevertheless, the literature suggests that most CD11c^+^CD11b^+^CD103^-^MHCII^+^ MPs in the small intestine are macrophages, and only a minority are DCs [25]. Because we were not able to use additional markers to clearly distinguish the DC and macrophage subpopulations within this category, we refer to them as MPs throughout. Much of the earlier literature on the role of CD11b^+^ MPs in inducing Th17 cells also predated this distinction. Future work will be required to further define which newly described subsets within this category are involved.

Phenotypic characterization of MPs in IL-15 KO and IL-15 Tg mice was carried out based on the expression of surface markers CD11b, CD8, and CD103. According to the literature, the tissue environment has a major impact on MP phenotypic and functional properties. Accordingly, no effects were seen in the spleen (data not shown). As shown in S3A Fig, when we first compared IL-15 Tg mice with their littermates by gating on MP subsets, a significant increase of CD103^+^ single positive MPs was found in the LP of IL-15 Tg mice (P<0.001) **(** S3B Fig **).** Although no significant difference in CD103^+^ MPs was observed in the LP of IL-15 KO mice compared to their WT counterpart, the trend was toward lower proportions in the KO, p = 0.16). In contrast, significantly lower proportions of CD11b^+^ single positive MPs (**S3C Fig**) and CD103^+^CD11b^+^ double positive MPs **(** S3A Fig **)** were found in LP of IL-15 Tg mice, and a correspondingly greater number of CD11b^+^ MPs in the IL-15 KO mice (S3A and S3C Fig, **p<0.05).** To facilitate this comparison, we examined the ratios of CD11b^+^ single positive MPs / CD103^+^ single positive MPs and of double positive MPs/CD103^+^ single positive MPs in the LP of individual mice (that is paired comparisons) among the gated CD11c^+^MHCII^+^ LP MPs studied in S3A–S3C Fig (ratio shown **in** S3D and S3E Fig, respectively). These ratios were chosen as ratios of each population known to contain Th17-inducing MPs over CD103 single positive MPs known not to induce Th17 cells. The ratios were substantially higher in IL-15 KO mice *vs* WT and significantly lower in IL-15 Tg *vs* IL-15 Tg WT littermates **(** S3D and S3E Fig), paralleling the Th17 distribution.

To examine these MP differences in more detail, we also purified the CD11c^+^MHCII^+^ MPs to characterize LP MPs and splenic MPs. As shown in **Fig 2A, 2B, 2D, 2E and 2F**, we noted the presence of CD11b^+^ CD103^+^ double positive MPs in the LP (**Fig 2A and 2D**) but not in the spleen (**Fig 2B**), as expected. In the LP of IL-15 Tg *vs* WT littermate (**Fig 2A**, left two panels), the proportion of CD103^+^CD11b^+^ double positive (**Fig 2D**), and CD11b^+^CD103^-^ MPs (**Fig 2F**) were significantly lower than in the WT littermate. Indeed, Fig 2D and 2F show extremely similar patterns, even though the overall frequencies are higher for the CD11b single positive cells in **Fig 2F**. In contrast, CD103^+^CD11b^-^ MPs were significantly higher in the LP of the Tg than that of the WT littermates (**Fig 2E**). The reverse was true in the LP of the IL-15 KO *vs* its WT control (**Fig 2A, 2D, 2E and 2F**). However, the phenotypic difference is more evident in the IL-15 Tg than IL-15 KO mice, which might be related to the high level of IL-15 in IL-15 Tg mice. Thus, both the CD11b^+^CD103^+^ double positive population and the CD11b^+^ CD103^-^ single positive population of MPs that contain the two major inducers of Th17 cells showed similar patterns with higher levels in LP of the IL-15 KO mice and lower in that of the IL-15 transgenic mice (**Fig 2D & 2F**), distinct from the relative ratios between strains of the CD103^+^ single positive population (**Fig 2E**). Moreover, the ratios of CD103^+^CD11b^+^ double positive cells and of CD103^-^CD11b^+^ single positive cells to the CD103^+^ single positive cells both show the same relative pattern (**Fig 2G and 2H**). These exactly parallel the ratios found in gated CD11c^+^ MHCII^+^ LP MPs (S3D and S3E Fig). In all cases, the ratio was substantially higher in IL-15 KO mice *vs* WT and significantly lower in IL-15 Tg *vs* IL-15 Tg WT littermates **(** S3D and S3E Fig, **Fig 2G and 2H),** which correlated nicely with the Th17 distribution in the LP of these strains. Although the absolute values of the means of these ratios was consistently higher in the purified single positive MP populations (Fig 2H vs S3E Fig), there was no significant difference between the ratios of any mouse strain between the gated and the purified populations, indicating the same conclusion. It is possible that the CD11b^+^CD103^-^ subset that is responsible for most of the effect is the newly described CCR2^+^ bone fide DCs, not macrophages, that express these markers but lack CD64 and F4/80 [33].

We included another surface markers CD8α for MP characterization. Interestingly, CD8α^+^ MPs were greatly increased in both LP (**Fig 2C**) and spleen (not shown) of IL-15 Tg mice. Moreover, double positive CD103^+^CD8α^+^MPs were higher in IL-15 Tg mice than WT mice, but no population of CD8α^+^CD11b^+^ MPs was found in either spleen or LP (i.e. these markers are not co-expressed in the same MPs and this population is not believed to exist). Together, these results suggest IL-15 could condition the phenotype of MPs in the intestinal LP.

### LP MPs from IL-15 Tg have lower capacity to induce antigen specific Th17 differentiation *in vitro*

**Fig 3:**
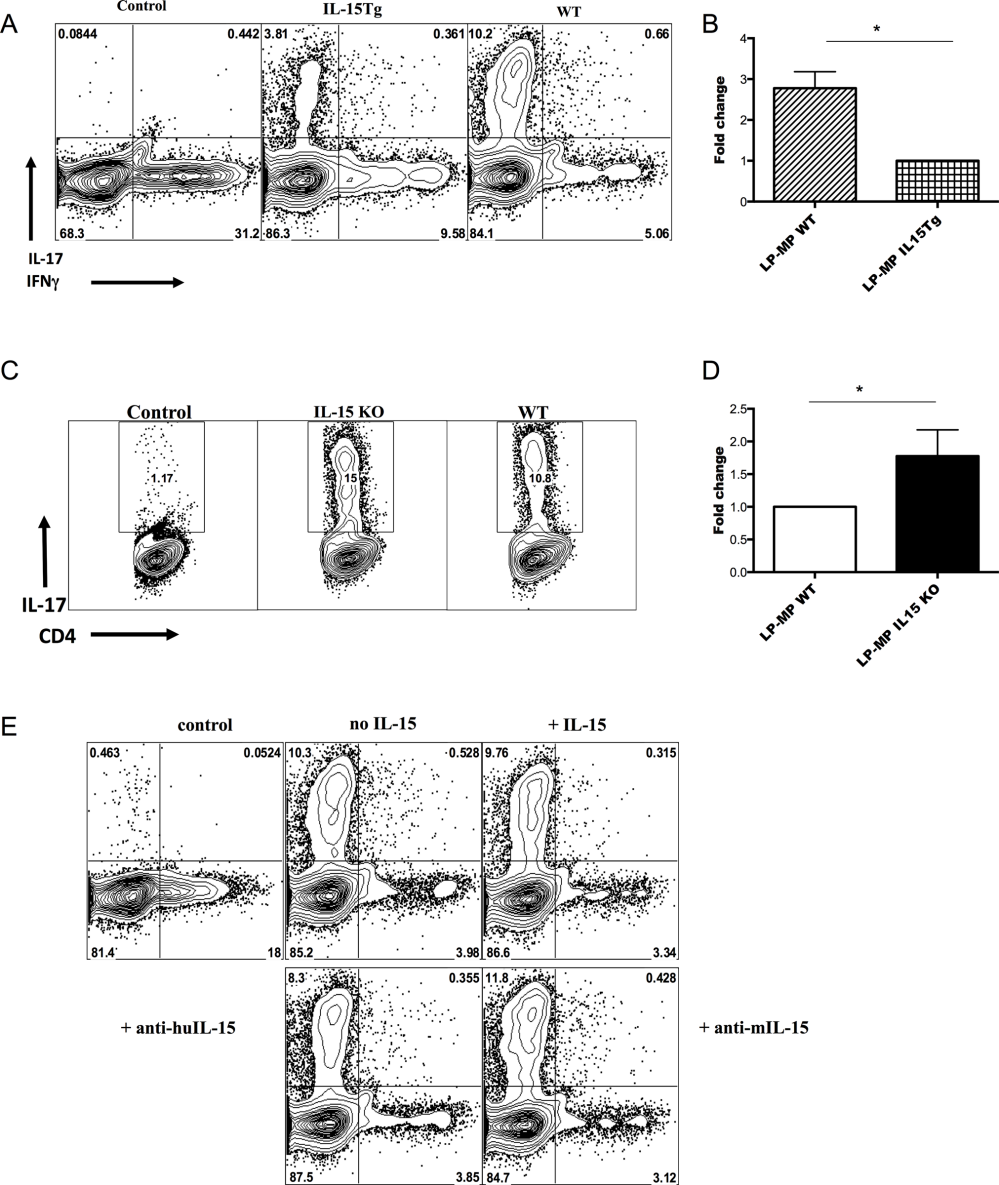
Flow cytometry of intracellular production of IL-17 by LP MPs from IL-15 Tg (A and B) or IL-15 KO (C and D) cocultured for 4 days with OTII T cells under Th17 conditions and re-stimulated for 4h with PMA and ionomycin. Data are representative of one of three independent experiments with comparable results. **A, B.** LP MPs from IL-15 Tg have lower capacity than WT to induce antigen specific Th17 differentiation *in vitro* (p = 0.0075 from Mann-Whitney test of 5 mice per group shown in B). **C, D.** LP MPs from IL-15 KO have higher capacity to induce antigen specific Th17 differentiation *in vitro* (p = 0.038 from Mann-Whitney test of 9 mice per group shown in D). B, D. The fold change of the induction of antigen specific Th17 relative to WT MPs is shown. For normalization, the induction of antigen specific of Th17 by LP MPs from WT was considered as 1 (*P<0.05 for N ≥5). E. Effect of adding or blocking IL-15 in the culture of OT II CD4^+^ T cells with WT LP MPs to induce Th17 cells. See Methods. Both anti-mouse IL-15 and anti-human IL-15, which react with mouse IL-15, were tested and are shown in the lower panels.

As mentioned before, according to the literature, the CD103^+^ MP subset helps the generation of Treg cells (but under inflammatory conditions can induce Th17), whereas the CD11b^+^ single positive and CD11b^+^CD103^+^ double positive MP subsets promote Th17 cell differentiation [25, 27, 38–42] [33]. Since we have noted distinct differences in the ratio of subsets of LP MPs from the IL-15 Tg mice and littermates, we next assessed the ability of the LP MPs to induce antigen specific Th17 differentiation in OVA-specific OTII CD4^+^ T cells *in vitro*. Purified MPs from small intestinal LP were pulsed with CD4 epitope OVA_323–339_ and used for antigen specific Th17 induction of OTII CD4^+^ T cells *in vitro* under Th17 polarizing conditions (TGF-β, IL-6, and anti-IFNγ). Purified LP MPs cultured together with OTII CD4^+^ T cells without polarizing conditions were used as a control. As shown in the **Fig 3A and 3B,** LP MPs from IL-15 Tg mice have lower capacity to induce antigen specific Th17 differentiation *in vitro* compared to those from WT mice, even under Th17-promoting conditions. Of note, OTII CD4^+^ T cells have a tendency to generate Th1 cells *in vitro* (as shown in the control). Among 5 such pairs of mice tested, the frequency of Th17 cells induced was almost 2.8-fold lower in the transgenic than the WT littermates (p = 0.0075) (**Fig 3B**) Consistent with these data, LP MPs from IL-15 KO mice conversely have higher capacity to induce antigen specific Th17 differentiation *in vitro* compared to those from WT mice (**Fig 3C and 3D**). Among 9 such pairs of mice tested, the frequency of Th17 cells induced was 1.8-fold higher in the IL-15 KO than the matched WT controls (p = 0.038 by Mann Whitney) (**Fig 3D**). (Unlike the case of IL-15 Tg mouse MPs, there was no apparent affect of the IL-15KO MPs on induction of Th1 cells producing IFNγ, data not shown.) Thus, the reciprocal pattern between IL-15 Tg and KO that was seen *in vivo* was recapitulated *in vitro* when the only cells from the Tg or KO LP were the MPs, and the OT II T cells were WT.

This finding directly implicates the source of MPs in determining the proportion of Th17 cells. However, the mechanism could depend on the ability or inability of the MPs to make IL-15 in the culture, or could be due to an indirect effect of the MP phenotypes we observed, especially the proportion of CD11b^+^ MPs. To distinguish these possibilities and test whether IL-15 was directly involved in Th17 differentiation, we directly added IL-15 or blocked it with anti-IL-15 antibodies in culture in the *in vitro* differentiation system, using all WT LP MPs and WT OTII cells. Addition or blockade of IL-15 failed to change antigen specific Th17 cell differentiation *in vitro* (**Fig 3E**). These data indicate that IL-15 does not directly affect Th17 differentiation at least *in vitro*, and the difference between the MPs from IL-15 KO or Tg mice is not just their ability to make IL-15. By exclusion, we conclude that the most likely scenario is that IL-15 is not acting directly on the CD4^+^ T cell but affects Th17 differentiation indirectly by affecting the proportion of CD11b^+^ and CD11b^+^CD103^+^ double positive MPs in the LP *in vivo*, which in turn promotes Th17 differentiation.

## Discussion

In contrast to CD8^+^ T cells, the effect of IL-15 on subsets of CD4^+^ T cells is not well understood. In our present study, significantly reduced frequencies of Th17 cells were observed in the small intestinal LP of IL-15 Tg mice, whereas these were increased in the LP of IL-15 KO mice. No differences in Th1 cells in the small intestinal LP were observed in these mice. The splenic results agree with an earlier report showing significantly increased IFNγ levels in the serum and increased CD8^+^ T cells expressing IFNγ in IL-15 Tg mice after infection with mycobacterium bovis bacillus Calmette-Guérin [61]. These results suggest that IL-15 plays an important role in type-1 cytokine development especially for CD8^+^ T cells. Although a reciprocal relationship between Tregs and Th17 cells is observed *in vitro* and in spleen cells in some knock out mice such as IL-6 KO [50], the frequencies of Tregs were found to be not significantly different in the small intestinal LP of IL-15 Tg mice compared to WT mice. Thus, this reciprocal developmental decision *in vivo* at the single-cell level must be more complicated and could be influenced by various local environmental factors [50].

The fact that the difference in Th17 frequency was found in the small intestinal LP and not in the spleens of IL-15 Tg or KO mice raised the question whether the difference could be due to differences in the intestinal microbiome in these mice. To exclude this possibility, we first used matched wild-type controls from the same colony as the Tg or KO mice, using wild-type littermates for the Tg animals, and second, co-housed the mice in the same cage with their WT controls to be sure that their intestinal flora were equilibrated among the strains. The co-housing (in the same cage) had no effect on the Th17 polarization observed, indicating that this was not the explanation of the findings. However, we expect that the lack of Th17 cells in the spleen compared to the intestine despite the greater ratio of CD11b^+^/CD103^+^ MPs in the spleen is probably due to the fact that commensal bacteria present in the intestine are needed to induce Th17, in addition to the effect any MP subsets. Indeed, it has been shown that most Th17 cells in the LP are specific for or induced by bacteria in the intestinal lumen, such as segmented filamentous bacteria [62, 63]. Thus, the appropriate MP subsets may be necessary (or more effective) but not sufficient to induce Th17 without other signals from bacteria [44]. In addition, the different MP subsets appear to have different growth factor, chemokine, and genetic requirements in different tissues that may also contribute to this difference [25–27, 43, 64, 65]. Also, vitamin A (retinol) from diet or from bile can imprint MPs in the intestinal mucosa [45].

MPs are thought to play a key role in maintaining the balance between tolerance and active immunity by discriminating between commensal microorganisms and potentially harmful pathogens. MPs in the intestine are abundant both in organized lymphoid organs such as the Peyer’s patches and the LP, where they act as sentinels [66]. MPs comprise several subsets, defined by expression of surface markers such as CD11b, CD8α, CD103, CD64, F4/80, CCR2, XCR1, SIRPα, and CD4, and these have distinct roles in the initiation of immunity to specific pathogens [25, 30, 33, 67, 68]. In our present study, the different MP subsets were characterized in IL-15 Tg mice and IL-15 KO mice according to the expression of surface markers, CD11b, CD8 and CD103.

Comparing the LP MPs with the splenic MPs, we observed that CD8α^+^CD11b^+^ double positive MPs were not found in either the spleen or the small intestinal LP, implying that these markers are mutually exclusive in MPs, consistent with the established literature. Moreover, a distinct CD11b^+^ CD103^+^ double positive MP population was found in the small intestinal LP but not in the spleen, most likely due to the fact that only a tiny number of CD103^+^ MPs were observed in the spleen. The different distribution of MP subsets in the spleen and LP indicated the importance of the local tissue microenvironment and opens the possibility that their balance could influence the outcome of T-cell priming.

CD103^+^ MPs in the LP could migrate to the mesenteric LNs in a CCR7 dependent manner and induce regulatory T cells via the dietary metabolite retinoic acid [34, 69]. Recently CD103^+^ MPs in the small intestinal LP were divided in to a small subset of CD103^+^ CD8α^+^ MPs and a large subset of CD103^+^ CD8α^-^ MPs. CD103^+^CD8 α^+^ LP MPs did not express the gene encoding retinoic acid-converting enzyme retinaldehyde dehydrogenase 2 (Raldh2) and were not involved in Foxp3^+^ Treg induction. CD103^+^CD8α^+^ LP MPs mainly induced antigen specific Th1 responses, and CTL activity *in vivo*, but not Th17 differentiation *in vitro* [70]. In our present study, CD103^+^CD8α^+^MPs, which were equivalent to CD11c^hi^CD11b^lo^ subset, were significantly higher in IL-15 Tg mice than WT mice. Consistent with this finding, we found lower frequencies of Th17 in the LP of IL-15 Tg mice.

In the LP, the CD11b^+^ MPs have been identified, some of which appeared to express tight junction proteins and could extend dendrites to sample luminal microbes in vitro [71]. These CX3CR1^+^ CD11b^+^ MPs that were originally thought to be DCs are now believed to be sessile macrophages deriving from Ly6C^+^ monocytes [25–27]. On the other hand, several subsets of MPs expressing CD11b in the LP can promote the induction of Th17 cell differentiation [25–28, 33, 37–42]. In our present study, significantly reduced CD11b^+^CD103^-^ MPs and CD11b^+^CD103^+^ MPs (that together contain the major Th17-inducing MPs) were observed in the LP of IL-15 Tg mice, whereas these MPs are conversely increased in the LP of IL-15 KO mice that have more Th17 cells. Moreover, the ratio of CD11b^+^ MPs / CD103^+^ MPs is higher in IL-15 KO mice *vs* WT and lower in IL-15 Tg *vs* their WT littermate mice, and this ratio is correlated with the higher frequencies of Th17 distribution in the LP. Thus, the distinct MP subset distribution in these mice could provide a possible mechanism for the higher frequency of Th17 in the LP *in vivo*.

To test this hypothesis, we asked whether IL-15 indirectly *via* an effect on MP subsets or directly constrains antigen specific Th17 differentiation *in vitro*. Here we used MPs purified from LP of IL-15 Tg mice, since marked MPs differences were found in these mice. Our data showed that LP MPs from IL-15 Tg mice were less effective at inducing antigen specific Th17 differentiation compared to those from WT mice. The converse was true for LP MPs purified from IL-15 KO mice. This result could be due to either the subset distribution of MPs in the LP of IL-15 Tg or KO mice that resulted from the higher or lower levels of IL-15 *in vivo*, or to a direct effect of more IL-15 in the culture made by the IL-15 Tg MPs, or lack thereof in the KO MPs. To distinguish these possibilities, we examined the direct effects of addition of IL-15 or blockade of IL-15 in WT MP-T cell co-cultures, and found that neither of these manipulations directly affected the induction of antigen specific Th17 cells *in vitro*. This result rules out one of the alternatives, the direct effect of IL-15 excess or deficiency on Th subsets. Moreover, the high frequencies of Th17 cells in small intestinal LP are correlated with the higher frequencies of CD11b^+^ MPs *in vivo* and the ratio of CD11b^+^ to CD103^+^ MPs. Taken together, these results suggest the interpretation that IL-15 might indirectly regulate Th17 polarization in the LP *via* its effect on MP subset differentiation in the LP, rather than through a direct effect of IL-15 on Th cell differentiation.

IL-15 and IL-2, as members of the common γ chain cytokine family, share many activities, but also have their own distinct functions. Similar to *Il2* ^*−/-*^ mice, IL-15 KO mice also have an increased frequency of Th17 cells in small intestinal LP. Moreover, addition or blockade of IL-2 *in vitro* could constrain or promote differentiation of IL-17, respectively [58]. However, in our present study, addition of IL-15 or blockade of IL-15 *in vitro* did not show any direct effect on the differentiation of antigen specific Th17 cells *in vitro*, although some effect of IL-15 on levels of IL-17A production by CD4^+^ T cells has been recently reported [72].

Increased expression of IL-15 has been reported in celiac disease and inflammatory bowel disease, which is critical in disease pathogenesis to induce proinflammatory cytokines, initiate epithelial apoptosis and trigger an anti-apoptotic pathway in human intraepithelial lymphocytes [73, 74]. Moreover, several distinct subpopulations of MPs in the human duodenal mucosa have been defined from celiac disease [75]. It might be interesting to further investigate the role of IL-15 in Th17 induction in the gut LP in a celiac disease model.

In conclusion, our data demonstrate that IL-15 plays a novel role in skewing the proportion of Th17 cells in the gut LP without affecting their proportion in other tissues tested, and without affecting the proportion of other major subsets of CD4^+^ T cells in the LP. This *in vivo* effect was mimicked *in vitro* when only MPs from the LP of these mice were used to differentiate WT Ova-specific CD4^+^ OTII cells in the presence of antigen and Th17-skewing conditions. Thus, LP MPs were implicated in this skewing. Because we could not find any direct effect of adding or blocking IL-15 in these cultures, we suggest that the *in vivo* effect is not likely due to the production or lack thereof of IL-15 by the LP MPs themselves, but more likely an indirect effect of variation in MP subset distribution in IL-15 Tg mice and IL-15 KO mice, consistent with the subset distribution we observed. We note that in vitro, IL-15 has been shown to skew the development of MPs from human monocytes to produce fewer of the CD11b^+^ cells [60], but we have not found a clear mechanism by which IL-15 causes this skewing. We hypothesize that involves differential sensitivity of these MP subsets or their precursors to either growth or inhibition by IL-15, perhaps due to receptor expression levels, during their maturation or recruitment to the tissue, but working out that mechanism is beyond the scope of this study. As IL-17-producing Th17 cells play an important role in the gut mucosal protection against bacteria, these studies may help to more effectively manipulate mucosal immunity for improving mucosal vaccination regimens, and may also explain certain disease pathologies.

## Supporting Information

S1 Fig Gating strategy and results obtained for staining with the CD3, CD4, IL17, Foxp3 and IFNγ staining. (PDF)

S2 Fig CD11c and MHCII double positive MPs. (PDF)

S3 Fig Effect of IL-15 transgene or KO on MP subsets in the LP, based on gating of MPs in unseparated populations. (PDF)

S1 Table ARRIVE checklist. (PDF)
