# Supplementary material for: Interleukin-15 Constrains Mucosal T Helper 17 Cell Generation: Influence of Mononuclear Phagocytes
Source: PLoS One. 2015 Nov 23;10(11):e0143001. doi: 10.1371/journal.pone.0143001 (PMC4658142; doi:10.1371/journal.pone.0143001)

Supplementary Figure 1. Gating strategy and results obtained for staining with the CD3, CD4, IL17, Foxp3 and IFNγ staining. Lymphocyte populations were gated based on SSC/FSC characteristics. Then CD3+ cell and CD4+ cells were respectively gated based on live cells. CD4+CD3+ cells were further analyzed for expression of IL17, Foxp3 and IFNγ .

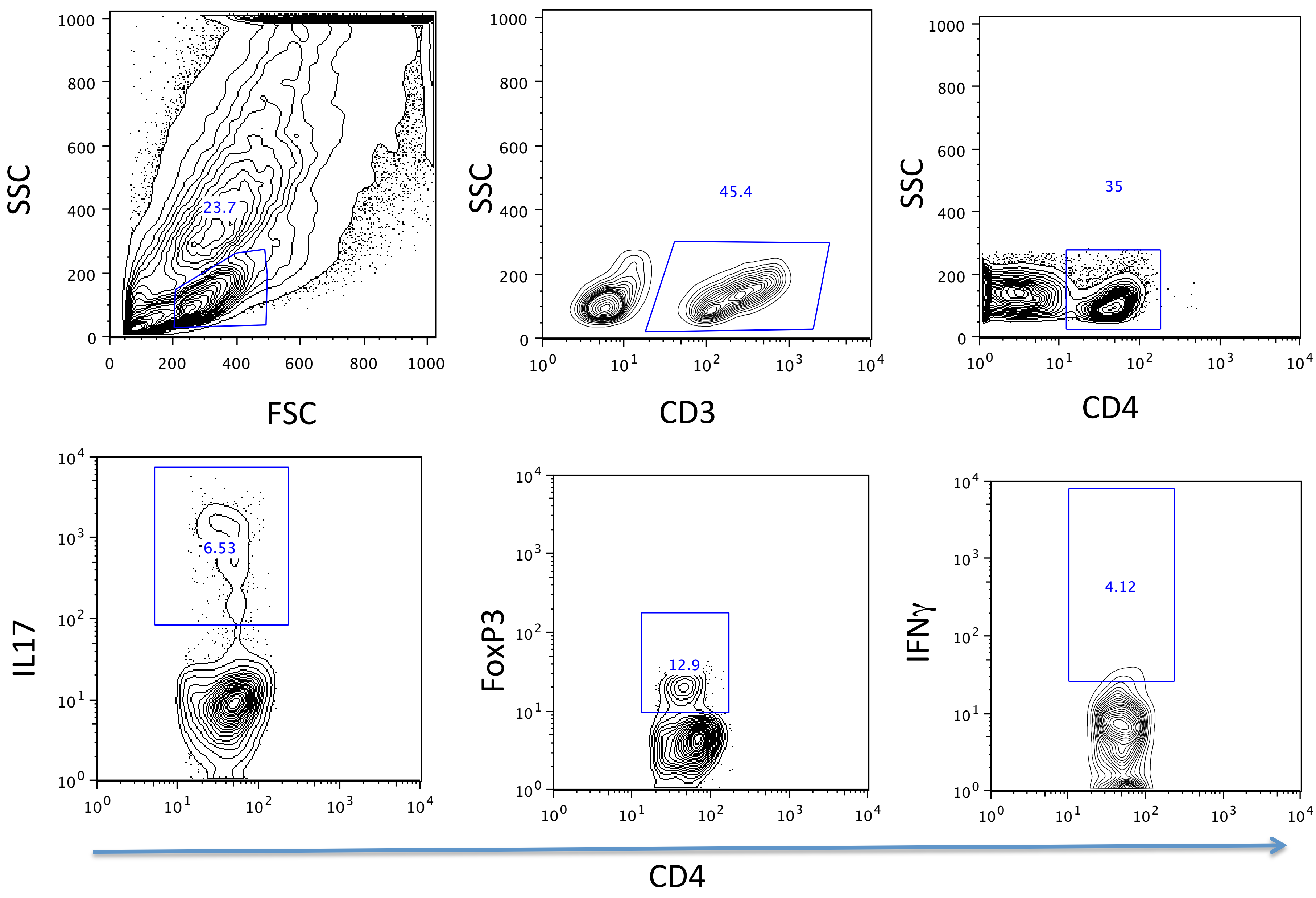

Supplement: S1 Fig — (PDF) [file pone.0143001.s001.pdf]
