# Supplementary figures and images for: Interleukin-15 Constrains Mucosal T Helper 17 Cell Generation: Influence of Mononuclear Phagocytes
Source: PLoS One. 2015 Nov 23;10(11):e0143001. doi: 10.1371/journal.pone.0143001 (PMC4658142; doi:10.1371/journal.pone.0143001)

Supplementary Figure 2. CD11c and MHCII double positive Dendritic cells.

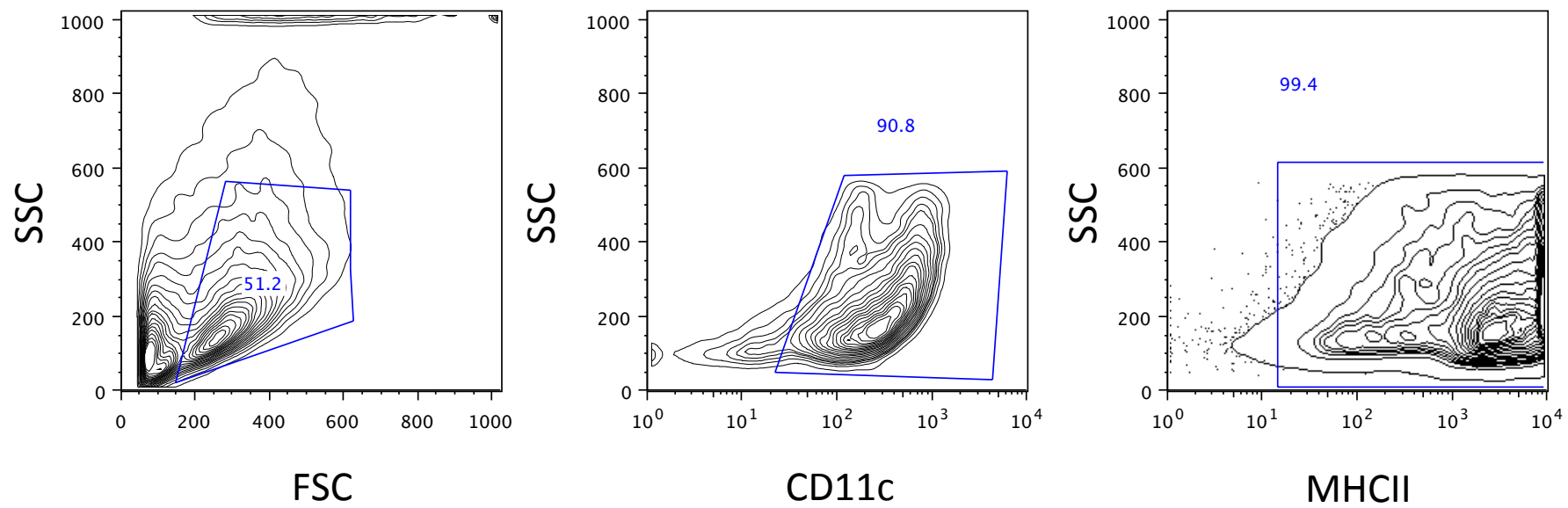

Supplement: S2 Fig — (PDF) [file pone.0143001.s002.pdf]
