# Supplementary material for: Interleukin-15 Constrains Mucosal T Helper 17 Cell Generation: Influence of Mononuclear Phagocytes
Source: PLoS One. 2015 Nov 23;10(11):e0143001. doi: 10.1371/journal.pone.0143001 (PMC4658142; doi:10.1371/journal.pone.0143001)

**Supplementary Fig. 3 Effect of IL-15 transgene or KO on MP subsets in the LP.** Cells were isolated from the small intestine and stained for surface makers CD11b, CD103, CD11c, and MHCII. **A-C.** Percentage of MPs expressing CD103, and/or CD11b in the small intestinal LP in IL-15 KO *vs* matched co-caged WT controls, and in IL-15 Tg *vs* co-caged WT littermates. **D-E.** The ratios of CD11b$^+$CD103$^+$/ CD103$^+$ MPs (D) and CD11b$^+$CD103$^-$/ CD103$^+$ MPs (E) based on gated whole LP cell preparations are shown. Paired Student t-tests were used for comparing different CD11b, CD103 and double positive subset MPs.

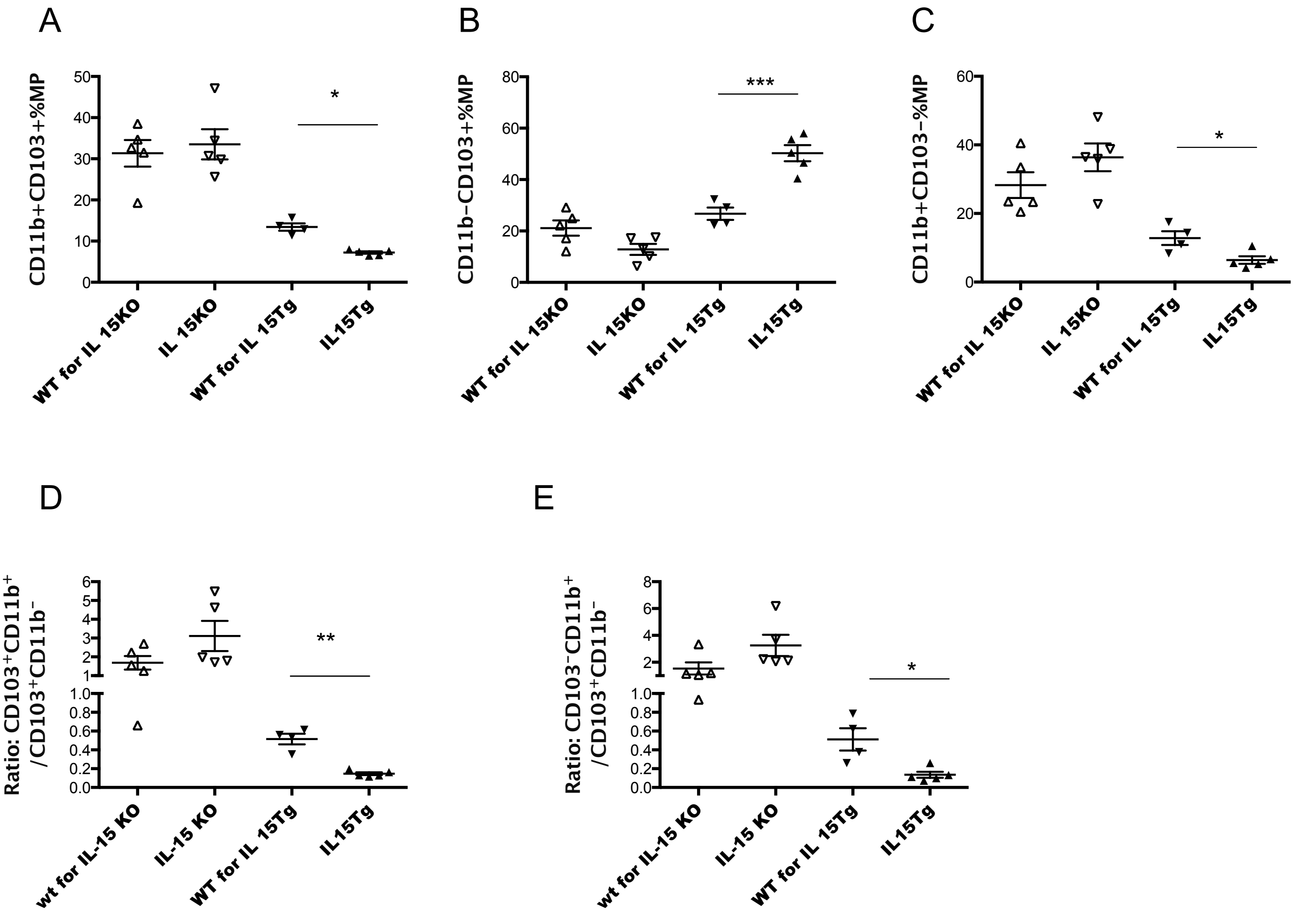

Supplement: S3 Fig — (PDF) [file pone.0143001.s003.pdf]
